# Relationship between heated tobacco product use and allergic rhinitis in Korean adults

**DOI:** 10.18332/tid/174130

**Published:** 2023-11-09

**Authors:** Young-Gyun Seo, Yu-Jin Paek, Joo-Hee Kim, Jwa-Kyung Kim, Hye-Mi Noh

**Affiliations:** 1 Department of Family Medicine, Hallym University Sacred Heart Hospital, Hallym University, Anyang, Republic of Korea; 2 Department of Internal Medicine, Division of Pulmonary, Allergy, and Critical Care Medicine, Hallym University Sacred Heart Hospital, Hallym University, Anyang, Republic of Korea; 3 Department of Internal Medicine & Kidney Research Institute, Hallym University Sacred Heart Hospital, Hallym University, Anyang, Republic of Korea

**Keywords:** smoking, tobacco use, heated tobacco products, allergic rhinitis

## Abstract

**INTRODUCTION:**

Combustible cigarette (CC) smoking is a risk factor for chronic obstructive pulmonary disease (COPD) and asthma, and some studies reported that tobacco smoking might affect the development or symptom control of allergic rhinitis, sinusitis, and atopic dermatitis. However, evidence on the health risks of heated tobacco products (HTPs) is lacking. We investigated the prevalence of respiratory and allergic diseases according to tobacco use types in Korean adults.

**METHODS:**

We used data from 18230 adults in the Korea National Health and Nutrition Examination Survey. Multiple logistic regression analyses were performed to assess the prevalence of respiratory and allergic diseases according to tobacco use types (current exclusive CC use, current exclusive HTPs use, and dual use of CC and HTPs).

**RESULTS:**

The prevalence of exclusive CC users, exclusive HTPs users, dual users of CC and HTPs was 15% (n=2740), 1% (n=182), and 2.4% (n=435), respectively. The prevalence of COPD was higher among past tobacco users (AOR=2.37; 95% CI: 1.02–5.51) versus no tobacco use group. The prevalence of asthma was higher among past tobacco users or exclusive CC users (AOR=1.73; 95% CI: 1.26–2.38, and AOR=1.57; 95% CI: 1.08–2.26) versus non-users of tobacco. The prevalence of allergic rhinitis was higher among past tobacco users versus non-users of tobacco (AOR=1.33; 95% CI: 1.13–1.57), and the prevalence of allergic rhinitis was higher among exclusive HTPs users versus non-users of tobacco or exclusive CC users (AOR=1.60; 95% CI: 1.06–2.42, and AOR=1.74; 95% CI: 1.14–2.66). The adjusted odds of sinusitis and atopic dermatitis were not significantly different between tobacco use types.

**CONCLUSIONS:**

Exclusive use of HTPs was associated with allergic rhinitis in Korean adults. Further longitudinal studies are needed to clarify the health risk of HTPs.

## INTRODUCTION

Combustible cigarette (CC) smoking is a risk factor for chronic obstructive pulmonary disease (COPD) and asthma^1,2^. Moreover, previous studies reported that tobacco smoking might affect the development or symptom control of allergic rhinitis, sinusitis, and atopic dermatitis^3-5^. Recently, tobacco companies developed heated tobacco products (HTPs), which contain an electrical heating component to heat tobacco leaves up to 350°C. Most toxicants and carcinogens of cigarettes are formed during combustion. HTP is also called a ‘Heat-not-Burn’ product, and it was marketed as reduced exposure to toxic materials compared with CC. In South Korea, the consumption of HTPs has increased rapidly since their launch in 2017, and, to date, they have been introduced in more than 50 countries worldwide^6^. HTPs appeal to youth and young adults because of their unique design and odorless nature^7^. Some studies reported that switching from CC to HTPs reduced harmful and potentially harmful compounds^8^; however, even some substances including propylene glycol, acetol, glycidol, and 2-propen-1-ol were higher in aerosol emitted HTPs than in CCs^9^. Further, aerosol emitted from HTPs contains tobacco-specific nitrosamines, which are one of the tobacco carcinogens^10^, and devices of HTPs release formaldehyde cyanohydrin at 90°C^11^. An in vitro study showed that HTPs caused lung damage and proinflammatory changes that were similar to the exposure to combustible cigarettes in mice^12^. In Japan, a case of acute eosinophilic pneumonia was reported, which developed after switching from CCs to HTPs^13^. To date, there is no evidence about the long-term health outcomes of HTPs. The World Health Organization (WHO) has stated that the marketing of HTPs should not be permitted until there is conclusive evidence of reduced health risks of HTPs compared with CCs^14^. Two recent Korean adolescent studies demonstrated the association between HTPs and allergic diseases^15,16^. Another concern of HTPs is dual use with other tobacco products such as CCs or e-cigarettes (ECs). Dual use is more common than exclusive HTPs use in adolescent smokers. According to Korea Youth Risk Behavior Survey 2019, the prevalence of exclusive HTPs use, dual use of CCs + HTPs, and ECs + HTPs were 1.5%, 8.6%, and 2.5%, respectively, among 4028 adolescent smokers^7^. The Korea Disease Control and Prevention Agency (KDCA) had investigated smoking prevalence through the Korea National Health and Nutrition Examination Survey (KNHANES) of Korean population, and it has included a questionnaire related to HTPs since 2018. In this regard, using KNHANES 2018–2020 data, we aimed to investigate: 1) the prevalence of exclusive HTPs use and dual use of CCs and HTPs; and 2) the prevalence of respiratory (COPD and sinusitis) and allergic diseases (asthma, allergic rhinitis, and atopic dermatitis) according to tobacco types in Korean adults.

## METHODS

### Study participants

The present study evaluated data from the KNHANES VII-3, VIII-1, and VIII-2, which is a nationally representative survey conducted between 2018 and 2020 by the KDCA. The KDCA selected 31261 individuals, and of the selected population, 23461 agreed to participate, yielding a response rate of 75%. This study included 19228 participants (aged ≥19 years) to evaluate the relationship between tobacco use type and respiratory and allergic diseases in Korean adults. We excluded participants who had missing survey records for smoking-related variables (n=998). Finally, 18230 participants were selected for the present study.

All procedures were performed in accordance with the Declaration of Helsinki, and signed informed consent was obtained from all KNHANES participants, and the KNHANES data are publicly available. KNHANES was approved by the KDCA Institutional Review Board (IRB: 2018-01-03-P-A, 2018-01-03-C-A, 2018-01-03-2C-A).

### Tobacco use type

Participants were asked the following questions:

1. Have you ever smoked more than 100 CCs in your lifetime?
2. Have you ever smoked CCs in the past 1 month?
3. Have you ever used HTPs in your lifetime?
4. Have you ever used HTPs in the past 1 month?

Participants who answered ‘no’ to first and third questions were categorized into ‘no tobacco use’. Participants who answered ‘yes’ to first and ‘no’ to second questions or ‘yes’ to third and ‘no’ to fourth questions were categorized into ‘past tobacco use’. Participants who answered ‘yes’ to first and second questions and ‘no’ to fourth question were categorized into ‘current exclusive CC use’. Participants who answered ‘yes’ to third and fourth questions and ‘no’ to second question were categorized into ‘current exclusive HTPs use’. Participants who answered ‘yes’ to first, second, third, and fourth questions were categorized into ‘current dual use’.

### Respiratory and allergic diseases

The prevalence of respiratory and allergic diseases was determined through the following questions:

- Have you ever been diagnosed with COPD by a doctor in your life?
- Have you ever been diagnosed with asthma by a doctor in your life?
- Have you ever been diagnosed with allergic rhinitis by a doctor in your life?
- Have you ever been diagnosed with sinusitis by a doctor in your life?
- Have you ever been diagnosed with atopic dermatitis by a doctor in your life?

### Other variables

The following data were collected as confounding variables: age, sex, education level (≤ elementary school, middle school, high school, or ≥ college), household income (quartile), alcohol intake (drinking alcohol at least once a month), adequate aerobic physical activity (moderate physical activity ≥150 min/week or vigorous physical activity ≥75 min/week), obesity status (body mass index ≥25 kg/m^2^), and comorbidities (defined by questionnaires regarding doctor-diagnosed chronic diseases such as hypertension, diabetes, or dyslipidemia)^17,18^.

### Statistical analysis

KNHANES data were extracted using two-stage stratified cluster sampling, therefore, the complex sampling weights are reflected in the data analyses^19^. To compare the general characteristics and the prevalence of respiratory and allergic diseases according to tobacco use type, we used one-way analysis of variance and χ^2^ tests. Multiple logistic regression analyses were performed to evaluate the prevalence of respiratory and allergic diseases according to tobacco use type. Adjusted odds ratios (AORs) and 95% confidence intervals (CIs) were calculated after accounting for potential confounding variables such as age, sex, education level, household income, monthly alcohol consumption, physical activity, obesity status, comorbidities (hypertension, diabetes, or dyslipidemia). All statistical analyses were conducted using Stata/MP version 14.0 (StataCorp.; College Station, TX, USA)^18^. All statistical tests were two-sided, and a p<0.05 was considered statistically significant.

## RESULTS

### General characteristics

Of the 18230 participants in the present study, 55.7% were women, and the mean age was 47.80 ± 0.24 years. Table 1 shows the general characteristics of the participants according to tobacco use type. The prevalence of current exclusive CC users, current exclusive HTPs users, dual users of CCs and HTPs was 15% (n=2740), 1% (n=182), and 2.4% (n=435), respectively. The highest proportion of men and the highest prevalence of hypertension, diabetes, and dyslipidemia were observed in past tobacco users. The highest proportion of highly educated participants (≥ high school) and the highest proportion of high-income participants were observed in current exclusive HTPs users. The youngest age, highest proportion of alcohol drinkers, highest proportion of participants who performed moderate to severe intensity physical activity, and highest proportion of participants with obesity were observed in current dual users.

**Table 1:** General characteristics of the participants by tobacco use type, South Korea, 2018–2020

*Characteristics*	*No tobacco use (N=11244) n (%)*	*Past tobacco use (N=3629) n (%)*	*Current exclusive CC use (N=2740) n (%)*	*Current exclusive HTPs use (N=182) n (%)*	*Current dual use (N=435) n (%)*	*p*
**Age** (years), mean ± SD	47.55 ± 0.28^b,c,d,e^	53.07 ± 0.35^a,c,d,e^	45.45 ± 0.35^a,b,d,e^	39.60 ± 0.71^a,b,c^	36.03 ± 0.55^a,b,c^	<0.001
**Sex**						<0.001
Male	2183 (19.41)	3127 (86.17)	2254 (82.26)	149 (81.87)	371 (85.29)	
Female	9061 (80.59)	502 (13.83)	486 (17.74)	33 (18.13)	64 (14.71)	
**Education level**						<0.001
≤Elementary school	2250 (21.08)	577 (16.88)	321 (12.48)	0 (0.00)	4 (0.94)	
Middle school	968 (9.07)	409 (11.97)	282 (10.96)	3 (1.69)	17 (4.01)	
High school	3373 (31.60)	1132 (33.12)	1132 (44.01)	61 (34.27)	172 (40.57)	
≥College	4084 (38.26)	1300 (38.03)	837 (32.54)	114 (64.04)	231 (54.48)	
**Monthly income**						<0.001
Q1	2647 (23.64)	860 (23.76)	870 (31.83)	33 (18.13)	106 (24.42)	
Q2	2754 (24.60)	906 (25.03)	737 (26.97)	38 (20.88)	107 (24.65)	
Q3	2848 (25.44)	922 (25.47)	615 (22.50)	54 (29.67)	119 (27.42)	
Q4	2947 (26.32)	932 (25.75)	511 (18.70)	57 (31.32)	102 (23.50)	
**Alcohol consumption**						<0.001
< once a month	6602 (58.73)	1243 (34.25)	674 (24.60)	31 (17.03)	61 (14.02)	
≥ once a month	4639 (41.27)	2386 (65.75)	2066 (75.40)	151 (82.97)	374 (85.98)	
**Physical activity** (min/week)						<0.001
Moderate <150 or vigorous <75	6210 (58.22)	1934 (56.63)	1535 (59.68)	92 (51.69)	195 (45.99)	
Moderate ≥150 or vigorous ≥75	4457 (41.78)	1481 (43.37)	1037 (40.32)	86 (48.31)	229 (54.01)	
**Body mass index** (kg/m^2^)						<0.001
<25	7532 (67.63)	2111 (58.66)	1700 (62.45)	99 (54.40)	227 (52.30)	
≥25	3605 (32.37)	1488 (41.34)	1022 (37.55)	83 (45.60)	207 (47.70)	
**Hypertension**	2739 (24.36)	1220 (33.62)	617 (22.52)	26 (14.29)	36 (8.28)	<0.001
**Diabetes**	1026 (9.13)	516 (14.22)	291 (10.62)	12 (6.59)	18 (4.14)	<0.001
**Dyslipidemia**	2311 (20.56)	843 (23.24)	486 (17.74)	22 (12.09)	40 (9.20)	<0.001

CC: combustible cigarette. HTPs: heated tobacco products. Post hoc analysis using one-way analysis of variance and χ^2^ tests.

a p<0.05 versus no tobacco use.

b p<0.05 versus past tobacco use.

c p<0.05 versus current exclusive CC use.

d p<0.05 versus current exclusive HTPs use.

e p<0.05 versus current exclusive dual use.

### Prevalence of respiratory and allergic diseases

Table 2 shows the prevalence of diseases according to tobacco use type. The prevalence of allergic rhinitis was the highest in current exclusive HTPs users (p<0.001), and the prevalence of allergic rhinitis was higher in current dual users (16%) and current exclusive HTPs users (25.28%) compared to current exclusive CC users (12.02%) (p=0.022 and p<0.001). The prevalence of atopic dermatitis was the highest in current dual users (p<0.001), and the prevalence of atopic dermatitis was higher in current dual users (6.59%) compared to current exclusive CC users (3.53%) (p=0.003).

**Table 2:** Comparison of the prevalence of diseases by tobacco use type, South Korea, 2018–2020

*Disease*	*No tobacco use (N=11244) n (%)*	*Past tobacco use (N=3629) n (%)*	*Current exclusive CC use (N=2740) n (%)*	*Current exclusive HTPs use (N=182) n (%)*	*Current dual use (N=435) n (%)*	*p*
**COPD**						0.033
No	5313 (99.57)	1995 (98.96)	1265 (99.29)	59 (100)	124 (100)	
Yes	23 (0.43)	21 (1.04)	9 (0.71)	0 (0)	0 (0)	
**Asthma**						0.076
No	10397 (97.05)	3297 (96.18)	2491 (96.63)	175 (98.31)	409 (96.24)	
Yes	316 (2.95)	131 (3.82)	87 (3.37)	3 (1.69)	16 (3.76)	
**Allergic rhinitis**						<0.001
No	9000 (84.03)	2968 (86.61)	2268 (87.98)	133 (74.72)	357 (84.00)	
Yes	1711 (15.97)	459 (13.39)	310 (12.02)	45 (25.28)	68 (16.00)	
**Sinusitis**						0.423
No	9954 (92.93)	3192 (93.14)	2413 (93.64)	164 (92.13)	388 (91.29)	
Yes	757 (7.07)	235 (6.86)	164 (6.36)	14 (7.87)	37 (8.71)	
**Atopic dermatitis**						<0.001
No	10327 (96.41)	3347 (97.67)	2487 (96.47)	169 (94.94)	397 (93.41)	
Yes	384 (3.59)	80 (2.33)	91 (3.53)	9 (5.06)	28 (6.59)	

CC: combustible cigarette. HTPs: heated tobacco products. COPD: chronic obstructive pulmonary disease.

### Multiple logistic regression for the prevalence of respiratory and allergic diseases by tobacco use types

**Figure 1:**
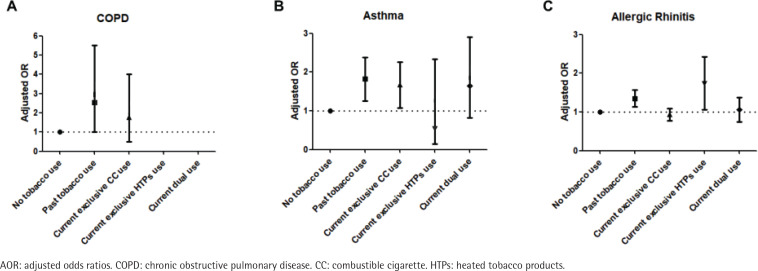
Adjusted odds ratios for COPD, asthma, and allergic rhinitis (A, B, C, respectively) compared to no tobacco use group

**Figure 2:**
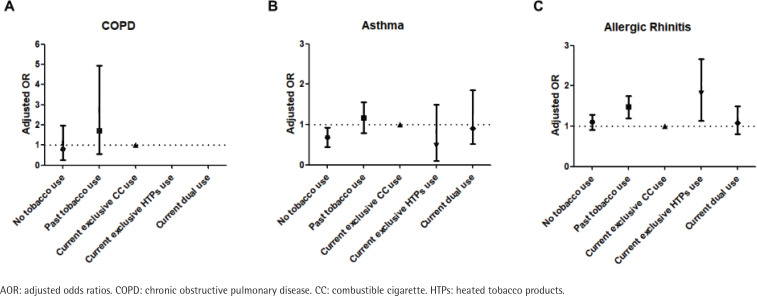
Adjusted odds ratios for COPD, asthma, and allergic rhinitis (A, B, C, respectively) compared to current exclusive CC use group

The results of logistic regression analyses for the prevalence of chronic respiratory diseases according to tobacco use type are shown in Table 3. After accounting for potential confounders, compared to non-users of tobacco, the past tobacco users had a higher prevalence for COPD (AOR=2.37; 95% CI: 1.02–5.51). Also, the past tobacco users or current exclusive CC users had a higher prevalence for asthma compared to non-users of tobacco (AOR=1.73; 95% CI: 1.26–2.38, and AOR=1.57; 95% CI: 1.08–2.26). The past tobacco users or current exclusive HTPs users had a higher prevalence for allergic rhinitis compared to non-users of tobacco (AOR=1.33; 95% CI: 1.13–1.57, and AOR=1.60; 95% CI: 1.06–2.42) (Figure 1). When we defined the current exclusive CC users as a reference group, the past tobacco users or current exclusive HTPs users had a higher prevalence for allergic rhinitis (AOR=1.45; 95% CI: 1.20–1.75, and AOR=1.74; 95% CI: 1.14–2.66) (Figure 2). The adjusted ORs of sinusitis and atopic dermatitis were not significantly different between tobacco use types.

**Table 3:** Multiple logistic regression for the prevalence of diseases by tobacco use type, South Korea, 2018–2020

	*COPD*	*Asthma*	*Allergic rhinitis*	*Sinusitis*	*Atopic dermatitis*
	*AOR (95% CI)*	*AOR (95% CI)*	*AOR (95% CI)*	*AOR (95% CI)*	*AOR (95% CI)*
**Model 1**					
No tobacco use (Ref.)	1	1	1	1	1
Past tobacco use	2.37 (1.02–5.51)	1.73 (1.26–2.38)	1.33 (1.13–1.57)	1.05 (0.85–1.29)	0.98 (0.69–1.38)
Current exclusive CC use	1.42 (0.50–4.00)	1.57 (1.08–2.26)	0.92 (0.78–1.09)	0.89 (0.70–1.13)	0.97 (0.72–1.32)
Current exclusive HTPs use	NA	0.59 (0.15–2.33)	1.60 (1.06–2.42)	0.87 (0.48–1.58)	1.21 (0.57–2.56)
Current dual use	NA	1.54 (0.82–2.90)	1.01 (0.74–1.37)	1.11 (0.73–1.68)	1.10 (0.65–1.85)
**Model 2**					
No tobacco use	0.70 (0.25–1.98)	0.64 (0.44–0.92)	1.08 (0.91– 1.29)	1.12 (0.89–1.42)	1.03 (0.76–1.40)
Past tobacco use	1.67 (0.56–4.95)	1.10 (0.79–1.55)	1.45 (1.20–1.75)	1.18 (0.93–1.49)	1.00 (0.71–1.42)
Current exclusive CC use (Ref.)	1	1	1	1	1
Current exclusive HTPs use	NA	0.38 (0.10–1.50)	1.74 (1.14–2.66)	0.97 (0.53–1.80)	1.24 (0.59–2.64)
Current dual use	NA	0.98 (0.52–1.86)	1.09 (0.80–1.50)	1.25 (0.81–1.92)	1.13 (0.69–1.87)

COPD: chronic obstructive pulmonary disease. CC: combustible cigarette. HTPs: heated tobacco products. AOR: adjusted odds ratio; adjusted for age, sex, education level, average monthly household income, monthly alcohol consumption, physical activity, obesity status, and comorbidities.

## DISCUSSION

In this large representative Korean study, current exclusive HTPs use was associated with higher odds of allergic rhinitis compared with non-users of tobacco and current exclusive CC users. There is no conclusive evidence on how HTPs affect the development of allergic rhinitis. However, recent studies revealed that HTPs contain unknown chemicals not found in CCs^20^, and even some harmful and potentially harmful chemicals (HPHCs), including propylene glycol, acetol, glycidol, and 2-propen-1-ol are significantly higher in HTPs than in CCs^9,11^. A previous study reported that short exposure (over 1 min) to propylene glycol caused ocular and upper airway symptoms, but not lower airway symptoms in healthy volunteers^21^. In addition, considering that occupational rhinitis caused by various chemicals such as chlorine, formaldehyde, etc., was often preceded by occupational asthma, we speculated that substances from HTPs could initially affect the upper airways and induce inflammation in the lower airways later^22,23^. This study showed that current exclusive HTPs use was associated with allergic rhinitis, but not bronchial asthma. However, future long-term experimental studies are needed to demonstrate the relationship between chronic exposure to aerosol emitted from HTPs and the development and symptoms of allergic rhinitis and asthma.

Recent studies with Korean adolescents, using the data from the Korea Youth Risk Behavior Survey 2018, demonstrated the association between HTPs and allergic diseases^18,19^. They reported that ever exclusive HTPs use was related to asthma, and dual use of HTPs and CCs was related to asthma and atopic dermatitis compared with non-use of tobacco. Moreover, triple-use of HTPs, CCs, and ECs was associated with asthma, atopic dermatitis, and allergic rhinitis^15^. In this study, current exclusive CC use and past tobacco use were associated with asthma compared with non-use of tobacco. Several studies have reported that current and former smokers had a higher risk of developing asthma^24,25^, which is linked to our study results. Meanwhile, we found no significant association between HTPs use and asthma in Korean adults. In Korea, HTPs were released in 2017; therefore, the relatively short period of use might not be sufficient to examine the impact of HTPs on asthma. The possible reason for the difference from previous adolescent research results might be the difference in prevalence and phenotypes of asthma in the age group of the study subjects. The incidence of allergic asthma is the highest in childhood, whereas the incidence of non-allergic asthma peaks in late adulthood^26,27^. Additionally, unlike previous adolescent studies that defined ‘ever’ use of HTPs, our study was limited to ‘current’ HTPs users. Further longitudinal studies are needed to clarify the association between HTPs use and asthma.

We found no association between tobacco use and atopic dermatitis in Korean adults. There have been mixed results regarding the relationship between tobacco use and atopic dermatitis. Some previous cross-sectional studies have reported that smoking is related to atopic dermatitis^4,28^; however, a recent large cohort study of US women reported no significant association between smoking and incident atopic dermatitis^29^.

In this study, past tobacco use was related to COPD, as already reported by several studies^2^. However, in our study subjects, none of the HTPs users had COPD, and we could not evaluate the association between HTPs use and COPD. A recent Korean nationwide cohort study evaluated the risk of non-combustible nicotine or tobacco products (NNTPs) use and COPD risk^30^. NNTPs included nicotine vaping products which vaporize nicotine-containing fluids and HTPs. They reported that dual users of CCs and NNTPs had a lower risk of COPD compared with continual CC users. However, long-term (≥5 years) CC quitters with NNTPs use increased the risk of COPD compared with long-term CC quitters without NNTPs use. Because they did not analyze HTPs separately from NNTPs, their results cannot be used to determine the impact of only HTPs use and the risk of COPD. An Italian study reported that switching to HTPs use from CCs for 3 years improved respiratory symptoms and exercise tolerance in COPD smokers^31^. Meanwhile, chronic respiratory symptoms (cough, phlegm, or congestion) for ≥3 months were more common in ever exclusive HTPs users and ever dual users than in ever exclusive CC users among Hong Kong youth^32^. Therefore, further long-term studies are needed to elucidate the impact of HTPs on the development of chronic respiratory diseases and symptom control.

### Strengths and limitations

To the best of our knowledge, this research is the first work that evaluated the association between HTPs use and respiratory and allergic diseases in Korean adults. In this study, current exclusive HTPs use was significantly associated with allergic rhinitis compared with non-users of tobacco and current exclusive CC users. We added evidence of HTPs use and allergic diseases.

However, this study has several limitations. First, due to the cross-sectional study design, a causal relationship between HTPs use and the risk of diseases was not guaranteed. Further longitudinal studies are necessary to confirm the impact of HTPs use and respiratory and allergic diseases. Second, our categorization of tobacco use type could not reflect the intermittent or fluctuating use of tobacco products. Also, since the data on the duration and the number of HTPs used were not available in the questionnaire, we could not assess the cumulative effects of HTPs and health outcomes. Third, despite the difference in HPHCs according to types of HTPs (IQOS, Glo, and Lil), data on the types of HTPs were not available in the questionnaire. It was not possible to analyze whether there were different associations with health outcomes by type of HTPs. Fourth, the diagnosis of respiratory and allergic diseases was not performed by medical doctors, and it was based on self-reported information by the study participants. This might lead to misclassification bias when participants incorrectly reported or are unaware of their health conditions. In addition, diagnoses were on a lifetime basis, which could be influenced by past health issues unrelated to tobacco use. Also, the data on the severity of the symptoms, and frequency of disease exacerbation, were not available. It was not possible to determine the course of the diseases according to the change in the tobacco products used. Fifth, despite the fact that we adjusted for a wide range of confounding variables, there might be unmeasured confounders such as occupational or environmental exposures affecting the prevalence of respiratory and allergic diseases. Finally, in this study, the relatively small number of HTPs users might limit statistical power. However, the sales of heated tobacco products have increased sharply from 363 million packs in 2019 to 540 million packs in 2022, and they accounted for 15% of all cigarette sales in South Korea^33^. Therefore, elucidating the health impact of HTPs is of great public health importance.

## CONCLUSIONS

Current exclusive HTPs use was associated with allergic rhinitis among Korean adults. Future studies on specific components of HTPs related to allergic rhinitis need to be conducted. Also, to clarify the health impact of HTPs, more longitudinal studies of HTPs on various health outcomes are needed.

## Data Availability

The data supporting this research are available from the following link: https://knhanes.kdca.go.kr/knhanes/main.do
